# Correction: Membranes linked by *trans-*SNARE complexes require lipids prone to non-bilayer structure for progression to fusion

**DOI:** 10.7554/eLife.08843

**Published:** 2015-05-27

**Authors:** Michael Zick, Christopher Stroupe, Amy Orr, Deborah Douville, William T Wickner

Zick M, Stroupe C, Orr A, Douville D, Wickner WT. 2014. Membranes linked by trans-SNARE complexes require lipids prone to non-bilayer structure for progression to fusion. *eLife* **3**:e01879. doi: 10.7554/eLife.01879

Published 4 March 2014

During preparation of the manuscript files, a systematic error was introduced in columns 2 and 3 of Table 1 (http://dx.doi.org/10.7554/eLife.01879.004), in which the numbers should read 10^4^ instead of 10^5^.

The corrected Table 1 is shown below:

Corrected Table 1

**Table:** 

Protein	Molar ratio of lipid: protein in RPL reactions*	Molar ratio of lipid: protein on vacuoles		Ratio (RPLs/vacuoles) of molar protein: lipid ratios in std. reactions†
		BJ3505	DKY6218	
Vam7p	2 × 10^3^	30 × 10^4^	6.5 × 10^4^	7 × 10^1^
Vam3p	2 × 10^3^	11 × 10^4^	22 × 10^4^	7 × 10^1^
Vti1p	2 × 10^3^	10 × 10^4^	13 × 10^4^	5 × 10^1^
Nyv1p	2 × 10^3^	4.3 × 10^4^	8.1 × 10^4^	3 × 10^1^
Ypt7p	4 × 10^3^	1.9 × 10^4^	1.8 × 10^4^	0.5 × 10^1^
Sec17p	7 × 10^3^	41 × 10^4^	13 × 10^4^	3 × 10^1^
Sec18p	1 × 10^3^	10 × 10^4^	13 × 10^4^	10 × 10^1^
Vps33p	6 × 10^3^	17 × 10^4^	31 × 10^4^	3 × 10^1^

The originally published version of Table 1 is also shown for reference:

Original Table 1

**Table:** 

Protein	Molar ratio of lipid: protein in RPL reactions*	Molar ratio of lipid: protein on vacuoles		Ratio (RPLs/vacuoles) of molar protein: lipid ratios in std. reactions†
		BJ3505	DKY6218	
Vam7p	2 × 10^3^	30 × 10^5^	6.5 × 10^5^	7 × 10^1^
Vam3p	2 × 10^3^	11 × 10^5^	22 × 10^5^	7 × 10^1^
Vti1p	2 × 10^3^	10 × 10^5^	13 × 10^5^	5 × 10^1^
Nyv1p	2 × 10^3^	4.3 × 10^5^	8.1 × 10^5^	3 × 10^1^
Ypt7p	4 × 10^3^	1.9 × 10^5^	1.8 × 10^5^	0.5 × 10^1^
Sec17p	7 × 10^3^	41 × 10^5^	13 × 10^5^	3 × 10^1^
Sec18p	1 × 10^3^	10 × 10^5^	13 × 10^5^	10 × 10^1^
Vps33p	6 × 10^3^	17 × 10^5^	31 × 10^5^	3 × 10^1^

The article has been corrected accordingly.

